# Editorial: Effects of Androgens on Immunity to Self and Foreign

**DOI:** 10.3389/fimmu.2020.630066

**Published:** 2020-12-21

**Authors:** Trine N. Jørgensen, Susan Kovats, Hanna Lotter

**Affiliations:** ^1^ Department of Inflammation and Immunity, Cleveland Clinic, Lerner Research Institute, Cleveland, OH, United States; ^2^ Arthritis and Clinical Immunology Research Program, Oklahoma Medical Research Foundation, Oklahoma City, OK, United States; ^3^ Department of Molecular Parasitology and Immunology, Bernhard Nocht Institute for Tropical Medicine (BMITM), Hamburg, Germany

**Keywords:** androgen, sex differences, immune cells, cancer, infection, autoimmunity

It is well established that male and female sex hormones regulate our immune system, leading to sex-specific differences in the outcome and manifestation of many diseases. As such, females often show superior protection against infections and some tumors and elaborate a better vaccination response due to immune-modulating effects of estrogens and progesterone, yet this enhanced immunity is coupled with an increased incidence of autoimmune diseases [reviewed in (1, 2)]. While anecdotally known since ancient times, only over the past couple of decades has the immunosuppressive effect of male sex hormones [testosterone, dihydrotestosterone (DHT)] been recognized. Testosterone and its most active metabolite DHT bind cytosolic or membrane-bound androgen receptors (ARs) to either directly or indirectly modulate gene transcription. ARs are expressed by many cells in the body including the majority of developing and some mature immune cells. Investigations of the role of testosterone in infectious diseases, autoimmunity and cancer have revealed direct effects of testosterone on immune cell development and function, with a general outcome of immunosuppression.

Although it remains questionable if sex hormones can be effectively used as therapeutic agents to treat diseases due to the many potential side-effects of such treatment, an understanding of testosterone-regulated cellular and molecular pathways may facilitate the development of innovative intervention strategies involving non-steroidal selective AR modulators that mediate tissue-specific activation of ARs (3), or the identification of cell-specific downstream signaling components that could be therapeutically targeted by gene manipulation strategies or small molecule inhibitors. In this article collection, authors from all over the world present new data and discuss the cellular and molecular impact of androgens in a variety of diseases including infections, cancer and autoimmunity.

In recognition of the potential of AR activity to modulate disease incidence and prevalence, the scientific community is actively investigating mechanisms by which testosterone regulates immune cells. For example, further investigations into the testosterone-driven positive regulation of Foxp3 and regulatory T cells, or into the skewing of CD4+ and CD8+ responses, may lead to the identification of specific drugs that can drive *de novo* development of T cell subsets for treatment of autoinflammatory diseases. The effect of androgens on T cells is reviewed in the setting of autoimmune liver diseases by Henze et al. Similarly, it has been shown that androgens regulate both myeloid cell differentiation and function. In this collection, a role for androgens in myeloid cell development and differentiation is shown by Consiglio and Golnick and Hreha et al., and reviewed in the setting of lupus by Jones and Jorgensen, while functionally, the production of testosterone-regulated lipid mediators by myeloid cells is discussed in the context of inflammation resolution by Pace and Wertz. We are hopeful that these and similar observations will drive *de novo* discovery of reagents capable of controlling testosterone-driven T cell and myeloid cell differentiation and trafficking patterns in infectious diseases, cancer and autoimmunity.

In addition to direct effect on immune cells, a role for androgens in driving tissue-specific differences in inflammation susceptibility are emerging. For example, Alves et al., present data describing testosterone’s ability to control vascular dysfunction in an inflammasome (NLRP3)-dependent manner, and Wilhelmson et al. present new data supporting a role for thymic epithelial cells in driving androgen-dependent early T cell development and the accumulation of recent thymic emigrant T cell populations in spleens and lymph nodes. Finally, Tuku et al. present data suggesting that the primary cytokine response to H1N1-driven lung infection is guided by androgens, driving sex differences observed among individuals infected with this strain of influenza. Thus, testosterone likely also affects key target non-immune cells in tissues susceptible to (auto)immune regulation, thus further amplifying the differential development of autoimmunity, cancer and infectious disease responses between men and women (reviewed by Ben-Batalla et al.).

As the medical field is driven toward personalized medicine and the availability of sex-specific genetic and biological data and tests rise in numbers, there is increasingly a need, but also the possibility, for identification of endogenous variability between individuals. Hundreds of genes coding for immune-related proteins differ between individuals, providing insight into the complexity of personalized medicine (4). Thus, it may not be surprising that our ability to mount and control immune responses has repeatedly been found to also differ between the sexes. As a consequence, different populations are known to be disproportionately affected by autoimmunity and cancer [reviewed in (5)], with the sex of individuals representing a significant variable.

In summary, further studies are needed in controlled environments for us to fully understand the complex regulation imposed by sex hormones. Still, with the knowledge on hand, the development of small-molecule agonists and antagonists capable of affecting selected downstream sex-dependent regulatory pathways, or target sex hormone receptors in selected cells or tissues, is of great importance for the field as we move toward the development of *de novo* therapeutic agents capable of lowering the severity of diseases presenting with a sex-bias.

## Author Contributions

All authors contributed equally to this publication. All authors contributed to the article and approved the submitted version.

## Conflict of Interest

The authors declare that the research was conducted in the absence of any commercial or financial relationships that could be construed as a potential conflict of interest.
